# Impact of the Use of Vinasse Sludge on Agricultural Soil and Surface Waters

**DOI:** 10.1002/wer.70559

**Published:** 2026-09-03

**Authors:** Wagner Tavares Dos Santos, Célsio Assane, Carlos Frederico de Souza Castro, Bruno de Oliveira Costa Couto

**Affiliations:** ^1^ Federal Institute of Education Science and Technology Goiano Rio Verde Goiás Brazil

**Keywords:** energy transition, environmental impacts, sugar‐energy sector, vinasse sludge, water preservation, water quality

## Abstract

Brazil is one of the world's largest sugarcane producers, and the sugar‐energy industry generates large volumes of by‐products such as vinasse and vinasse sludge, which require appropriate management to minimize environmental impacts. This study evaluated the agricultural disposal of sludge generated during the cleaning of vinasse storage reservoirs and its potential effects on adjacent surface water quality in Costa Rica, Mato Grosso do Sul, Brazil. Surface water quality was monitored at eight sampling points between 2020 and 2023, covering dry and rainy seasons. A total of 34 physicochemical and microbiological parameters were analyzed, and statistical tests were applied to assess spatial and seasonal variations and differences between areas with and without sludge application. The results showed significant spatial differences for calcium, electrical conductivity, dissolved iron, magnesium, manganese, ammoniacal nitrogen, and pH, while seasonal differences were observed for turbidity, total chromium, dissolved iron, and pH. Higher concentrations of several parameters were recorded at points P6 and P7; however, all monitored variables remained within the limits established by CONAMA Resolution No. 357/2005. Comparisons between areas with and without sludge application did not indicate measurable deterioration of surface water quality associated with sludge disposal. These findings suggest that the agricultural use of vinasse sludge, when conducted in accordance with environmental regulations and appropriate management practices, can be environmentally safe. Nevertheless, long‐term monitoring remains essential to detect potential cumulative effects and ensure the sustainable management of water resources.

## Summary

- Controlled soil application of vinasse sludge did not significantly affect adjacent surface water quality over 4 years of monitoring.
- Long‐term, multiparameter monitoring is essential to detect potential impacts of agro‐industrial residues on water resources.
- Proper sludge management and regulatory compliance enable safe reuse within circular economy practices in the sugar‐energy sector.
- Seasonal variability influences some water quality parameters, reinforcing the need for dry and rainy period assessments.

## Graphical abstract

Based on 4 years of monitoring and multivariate statistical analysis of 34 physicochemical and microbiological parameters, this study demonstrates that the regulated application of vinasse tank sludge to soil did not promote statistically significant alterations in adjacent surface water quality under continuous environmental control.

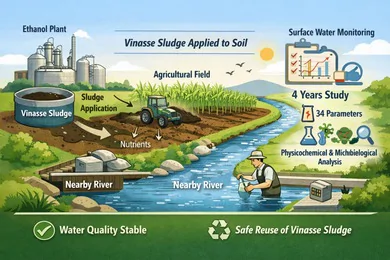

## 1 Introduction

Sugarcane is one of the most important agricultural commodities worldwide and accounts for approximately 80% of global sugar production. In addition to its role in food production, sugarcane is a strategic feedstock for bioethanol generation, contributing significantly to the global transition toward renewable energy sources (FAO 2015; European Commission – Directorate‐General for Agriculture and Rural Development 2025). Brazil is the world's largest producer of sugarcane and the second‐largest producer of ethanol, making the sugar‐energy sector a key component of its economy and energy matrix (Dos Santos et al. 2020; FAO 2021).

Despite its environmental benefits as a renewable energy source, ethanol production generates substantial quantities of by‐products and residues that require appropriate management to prevent adverse environmental impacts. Among these by‐products, vinasse is the most abundant liquid residue generated during the fermentation and distillation processes. On average, between 9 and 14 L of vinasse are produced for every liter of ethanol generated (Silva 2017). Vinasse is characterized by high concentrations of organic matter, nitrogen (N), phosphorus (P), potassium (K), and other dissolved compounds, as well as elevated biochemical oxygen demand (BOD) and chemical oxygen demand (COD) (Santos et al. 2017; Stephen et al. 2024).

Due to its high nutrient content, vinasse is widely used in agricultural systems, particularly through fertigation practices, where it serves as a source of water and nutrients for crops (Mahapatra et al. 2024). However, when improperly managed, excessive applications may alter soil chemical properties and increase the risk of nutrient leaching, salinization, eutrophication, and contamination of surface and groundwater resources (Carrilho et al. 2016; Fuess 2017). Similar concerns apply to vinasse sludge, a sedimented material composed of organic matter, sugarcane residues, and mineral particles that accumulates at the bottom of storage reservoirs.

Although vinasse is routinely applied through fertigation in the study area, the present investigation specifically evaluated the environmental implications associated with the agricultural application of vinasse sludge removed from storage reservoirs. Therefore, potential impacts associated exclusively with vinasse fertigation were beyond the scope of this study.

In modern sugar‐energy plants, vinasse is commonly stored in large reservoirs lined with high‐density polyethylene (HDPE) membranes before being distributed to agricultural fields through fertigation systems (Lavoie et al. 2013). During storage, suspended solids gradually settle, forming vinasse sludge. Periodic cleaning of these reservoirs is required to maintain operational efficiency and storage capacity. The resulting sludge contains nutrients and organic matter that may provide agronomic benefits when incorporated into the soil. Nevertheless, inadequate handling, transportation, or disposal of this material may increase the risk of soil degradation and contamination of nearby water bodies through runoff, erosion, and nutrient transport processes.

Several studies have demonstrated that appropriate treatment and management can transform vinasse and its sludge from potential environmental liabilities into valuable agricultural inputs, contributing to nutrient recycling, soil fertility improvement, and circular economy strategies (Glória and Orlando Filho 1983; Rossetto 1987; Utami et al. 2020; Calderón et al. 2025). However, information remains limited regarding the long‐term environmental effects of vinasse sludge disposal on agricultural soils and adjacent surface waters, particularly under field conditions in large‐scale sugarcane production systems.

Therefore, this study aimed to evaluate the environmental suitability of applying vinasse reservoir sludge to agricultural soils and to assess its potential impacts on nearby surface water quality through a 4‐year monitoring program involving physical, chemical, and microbiological analyses conducted at eight sampling points located within the area of influence of a sugar‐energy production system in Costa Rica, Mato Grosso do Sul, Brazil.

## 2 Materials and Methods

### 2.1 Study Area

The study was carried out in the municipality of Costa Rica, Mato Grosso do Sul, Brazil. The municipality has approximately 26,000 inhabitants, an area of 4159 km^2^, and an average altitude of 641 m (IBGE 2022). The regional climate is classified as humid subtropical with dry winter and hot summer (Cwa), according to the Köppen classification, characterized by a marked seasonality with a rainy season extending from October to April and a dry season from May to September. Mean annual precipitation ranges between 1500 and 1800 mm, with most rainfall concentrated during the summer months.

The study area is located within the upper portion of the Sucuriú River watershed, an important tributary of the Paraná River Basin (AMBDATA 2024). The Sucuriú River plays a strategic role in regional water supply, irrigation, agricultural production, and hydroelectric power generation. The monitored streams evaluated in this study are tributaries of the Sucuriú River and drain predominantly agricultural landscapes characterized by extensive sugarcane cultivation, cattle ranching, and native Cerrado vegetation remnants.

**FIGURE 1:**
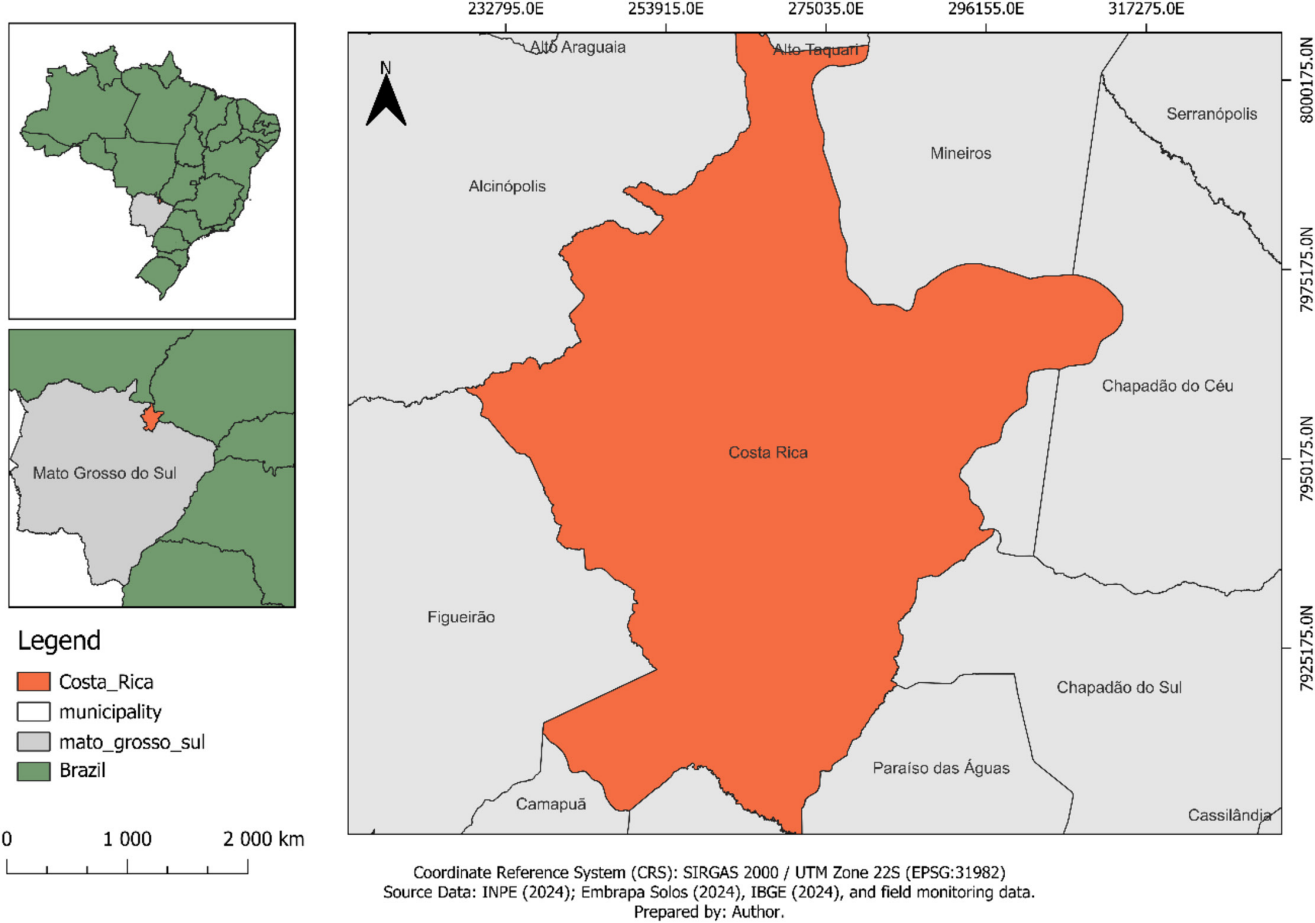
Location of the study area.

The predominant soils are Dystrophic Red Latosols (LVd3 and LVd9) and Orthic Quartzarenic Neosols (RQo10) (Figure 1), according to the Brazilian Soil Classification System. Red Latosols are highly weathered soils rich in iron and aluminum oxides, presenting high infiltration capacity and significant nutrient adsorption potential (Ker 1997). In contrast, Quartzarenic Neosols are sandy soils with low natural fertility and reduced capacity to retain nutrients, making them potentially more susceptible to nutrient leaching and sediment transport.

### 2.2 Surface Water Monitoring Network

**FIGURE 2:**
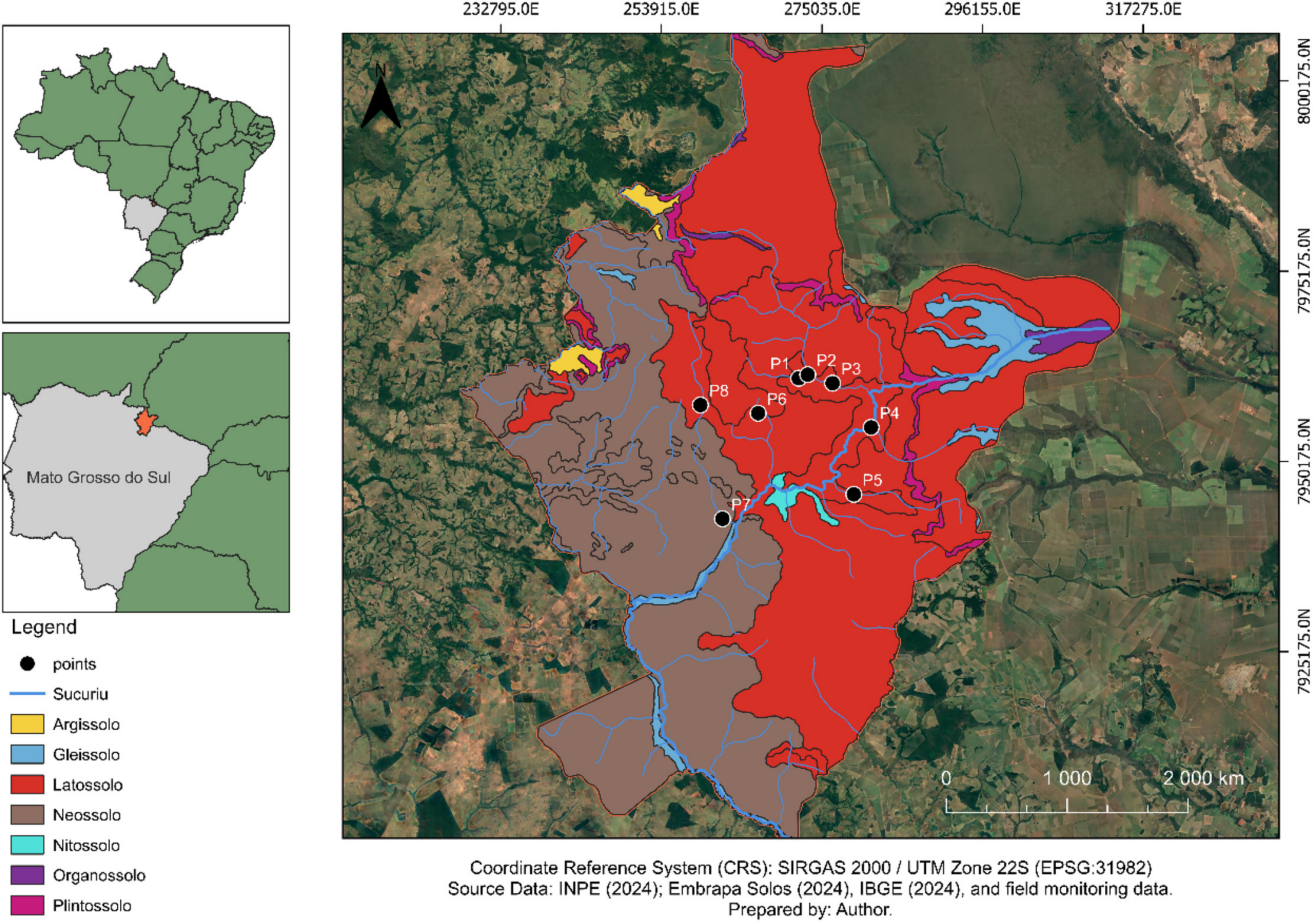
Monitoring points and their relationship to soil classification.

A monitoring network composed of eight sampling points (P1–P8) was established to evaluate the potential influence of vinasse sludge application on surface water quality within the study area (Figure 2). The selection of monitoring sites considered their proximity to sludge application areas, hydrological connectivity within the watershed, accessibility for sampling, and the need to include reference locations unaffected by sludge disposal activities.

All monitored watercourses are tributaries of the Sucuriú River, one of the main rivers in the region, and drain areas characterized predominantly by sugarcane cultivation and other agricultural activities. The monitoring network was designed to assess both local and cumulative effects of vinasse sludge management on water quality at the watershed scale.

The geographic coordinates and characteristics of each monitoring point are presented in Table 1. Points P1 (Indaiá Stream upstream of the water intake), P2 (Baús Stream at the water intake), P3 (Baús Stream downstream of the water intake), P4 (Tributary of the Sucuriú River 1), P5 (Cascavel Stream downstream), and P6 (Tributary of the Sucuriú River 2) are located in areas influenced by agricultural activities where vinasse sludge has been applied for approximately 8 years. These sites were selected because of their proximity to application areas and their greater susceptibility to potential changes in water quality resulting from nutrient and sediment transport processes.

**TABLE 1:** Monitoring points and geographic coordinates.

Points	Location	Geographic coordinates		Sludge application
		Latitude	Longitude	
01	Indaiá Stream upstream of the water intake	18°25′40.69″ S	53°09′32.05″ O	Yes
02	Baús Stream at the water intake	18°25′25.70″ S	53°08′51.73″ O	Yes
03	Baús Stream downstream of the water intake	18°26′03.63″ S	53°07′00.82″ O	Yes
04	Tributary of the Sucuriú River 1	18°28′08.10″ S	53°12′35.67″ O	Yes
05	Cascavel Stream—downstream	18°35′37.41″ S	53°15′21.52″ O	Yes
06	Tributary of the Sucuriú River 2	18°29′14.32″ S	53°04′10.23″ O	Yes
07	Tributary of the Sucuriú River 3	18°33′59.67″ S	53°05′31.34″ O	No
08	Cascavel Stream—upstream	18°27′30.01″ S	53°16′54.25″ O	No

*Source:* Author (2024).

Points P7 (Tributary of the Sucuriú River 3) and P8 (Cascavel Stream upstream) were selected as reference sites because they are located outside the direct influence of vinasse sludge application areas. These points provide baseline conditions for comparison with the potentially impacted sites and allow the distinction between natural variability and possible effects associated with sludge management practices.

Particular attention was given to P7 due to its location downstream of areas influenced by multiple land uses within the watershed. Although this site is not directly associated with sludge application, it may reflect cumulative impacts from agricultural and other anthropogenic activities occurring upstream. In contrast, P8 is located upstream of the Cascavel Stream and represents one of the least disturbed conditions within the monitoring network.

**FIGURE 3:**
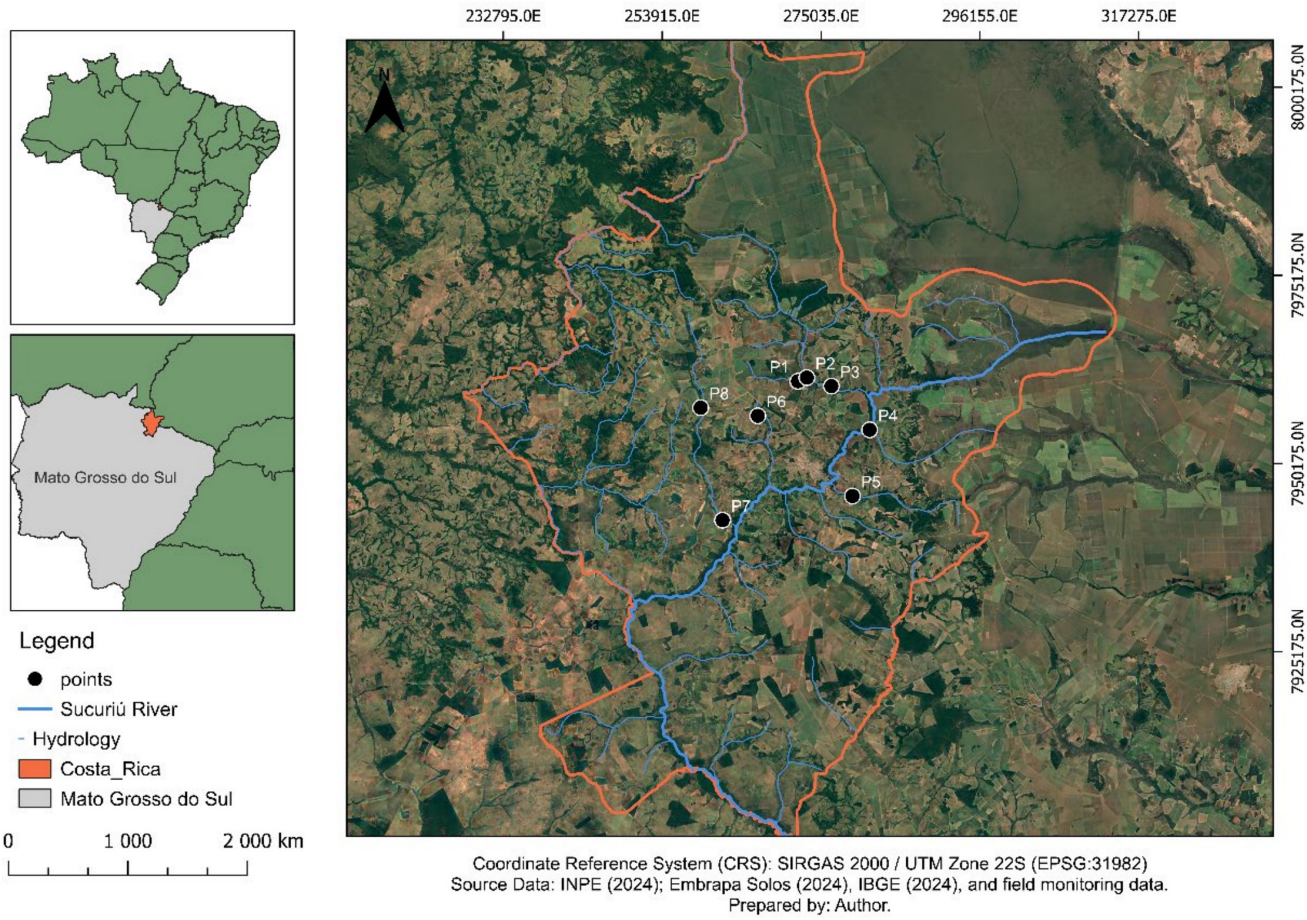
Monitoring points map and sludge application site. *Source:* Author (2024).

The spatial distribution of the monitoring points in relation to the watershed, soil classes, and sludge application areas is presented in Figures 2 and 3. This monitoring design enabled the comparison of water quality among sites with different levels of exposure to agricultural management practices and provided a comprehensive assessment of the potential environmental effects of vinasse sludge application on surface water resources.

### 2.3 Vinasse Sludge Removal and Agricultural Application

**FIGURE 4:**
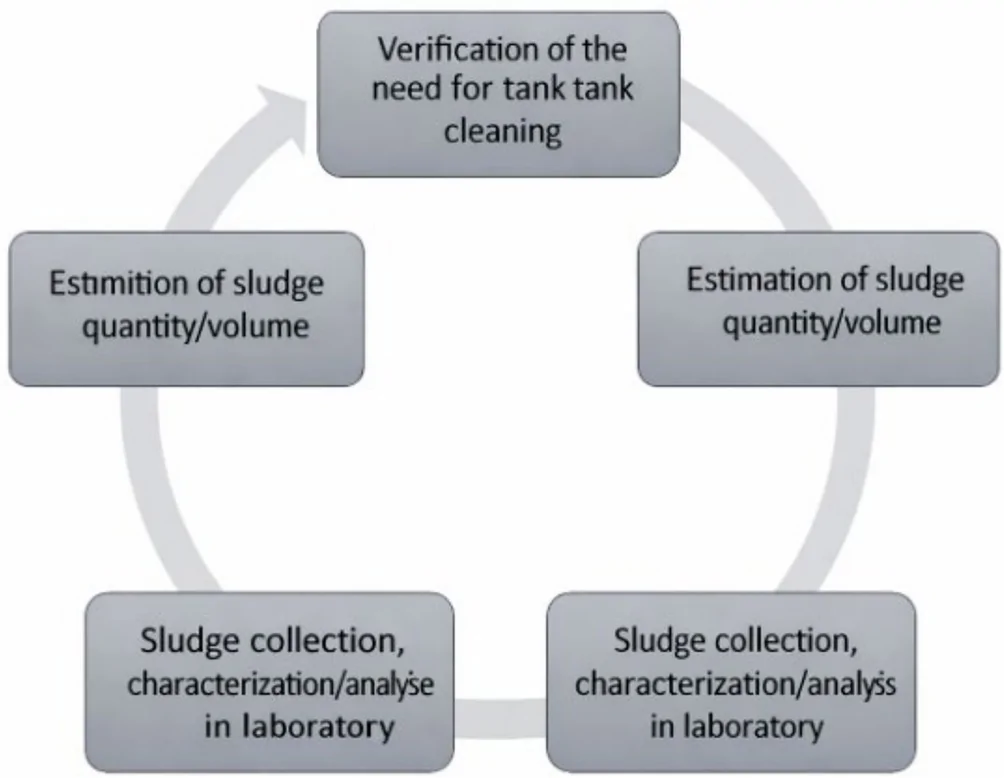
Flowchart of cleaning and incorporation of vinasse tank sludge into the soil. *Source:* Author (2024).

The removal and agricultural application of vinasse sludge followed the technical procedures established by the sugar‐energy plant and the environmental guidelines of SEMADESC Resolution No. 001/2023. The overall workflow adopted for sludge management is presented in Figure 4.

Initially, the sludge accumulated at the bottom of the vinasse storage reservoir was quantified to estimate the volume available for removal and agricultural use. The disposal reservoir (DR) had a total storage capacity of 1500 m^3^ and was approximately 70% occupied by accumulated sludge, corresponding to an estimated volume of 1050 m^3^. This estimate was obtained based on measurements of reservoir dimensions and the depth of the deposited sludge layer.

**FIGURE 5:**
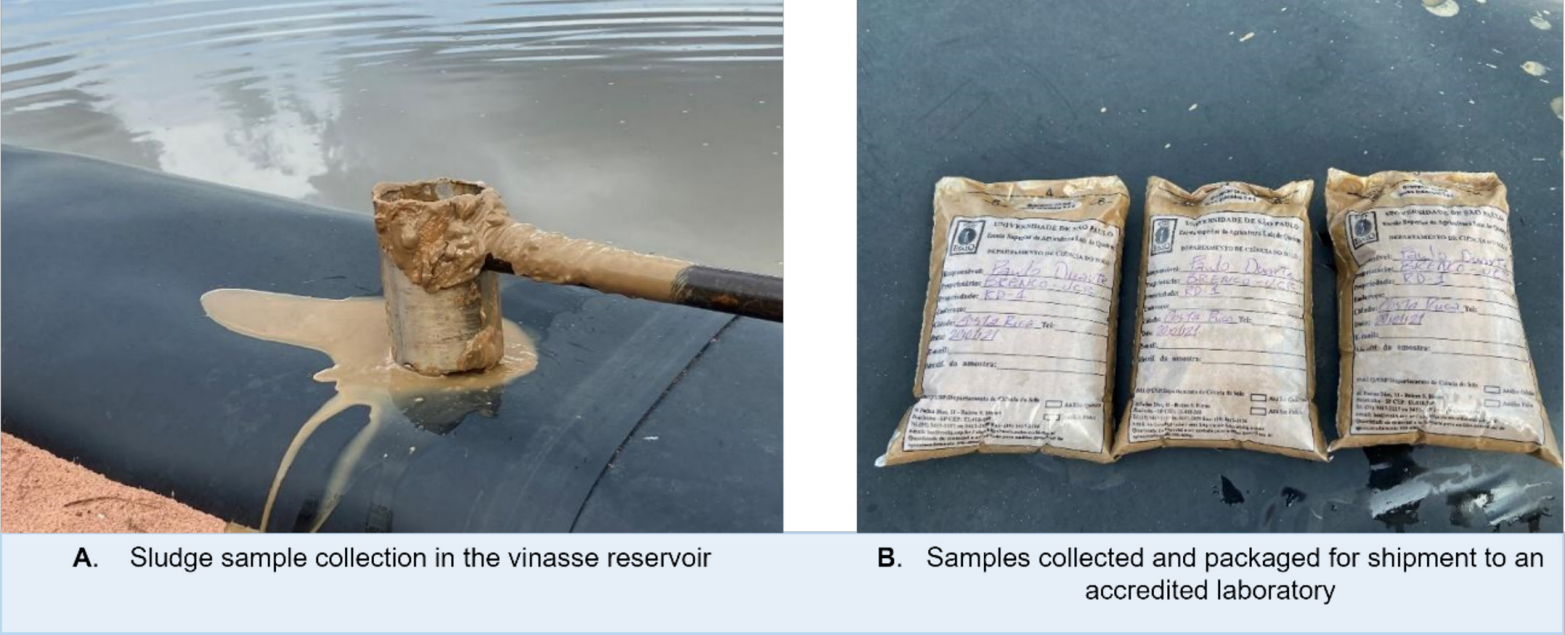
Collection, packaging, and preparation of sludge samples from the vinasse reservoir for submission to an accredited laboratory. *Source:* Author (2021).

Following volume estimation, sludge samples were collected directly from the reservoir for physicochemical characterization. The sampling procedure included collection, packaging, preservation, and transportation of samples to an accredited laboratory for analysis (Figure 5A,B). The laboratory analyses followed the requirements established by SEMADESC Resolution No. 001/2023 and aimed to determine the concentrations of macro and micronutrients, as well as other physicochemical properties relevant to agricultural use and environmental assessment.

**FIGURE 6:**
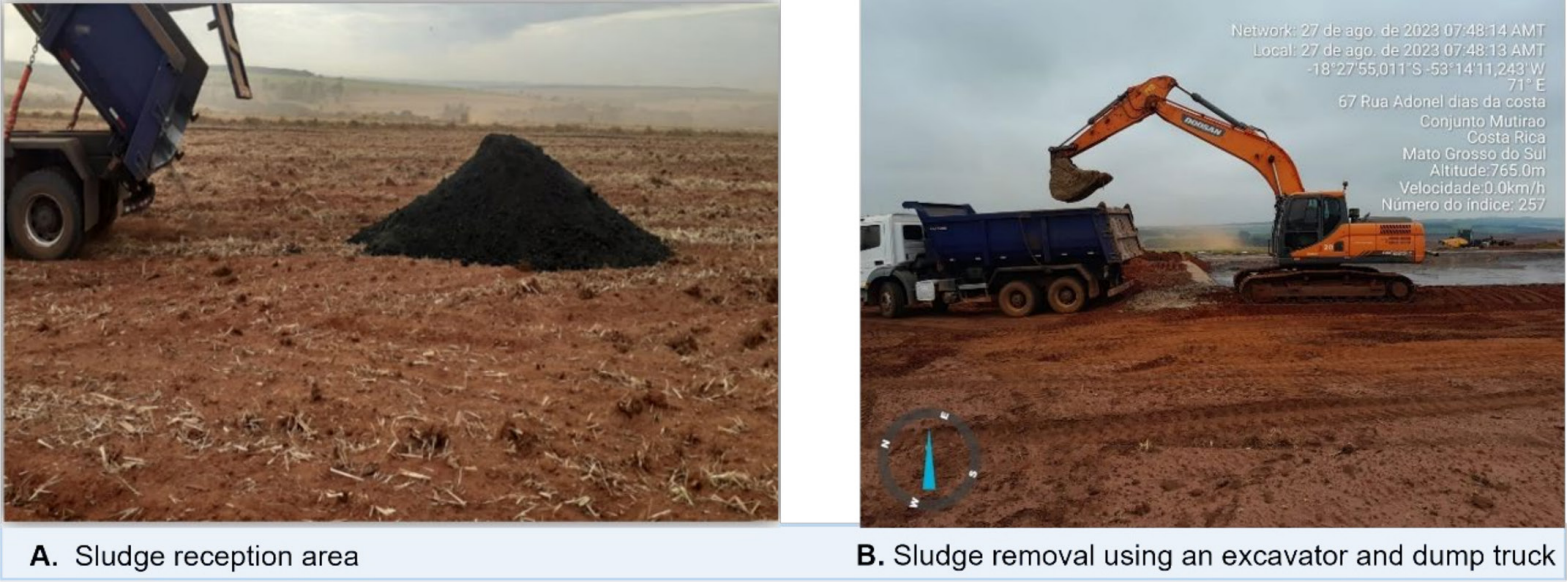
Sludge receiving site and removal procedure using an excavator and dump truck. *Source:* Author (2024).

Based on the analytical results and the estimated sludge volume, a receiving area was selected for agricultural application. The selected site consisted of a sugarcane field undergoing renovation prior to replanting, providing favorable conditions for sludge incorporation into the soil (Figure 6A). The choice of this area considered the compatibility between the sludge volume, nutrient content, soil characteristics, and operational feasibility.

Before sludge removal, the reservoir was drained to the maximum extent possible. The accumulated material was then excavated using hydraulic equipment positioned outside the reservoir and loaded into dump trucks for transportation to the designated application area (Figure 6B). This procedure minimized disturbance to the reservoir structure and reduced the risk of accidental releases during the removal process.

**FIGURE 7:**
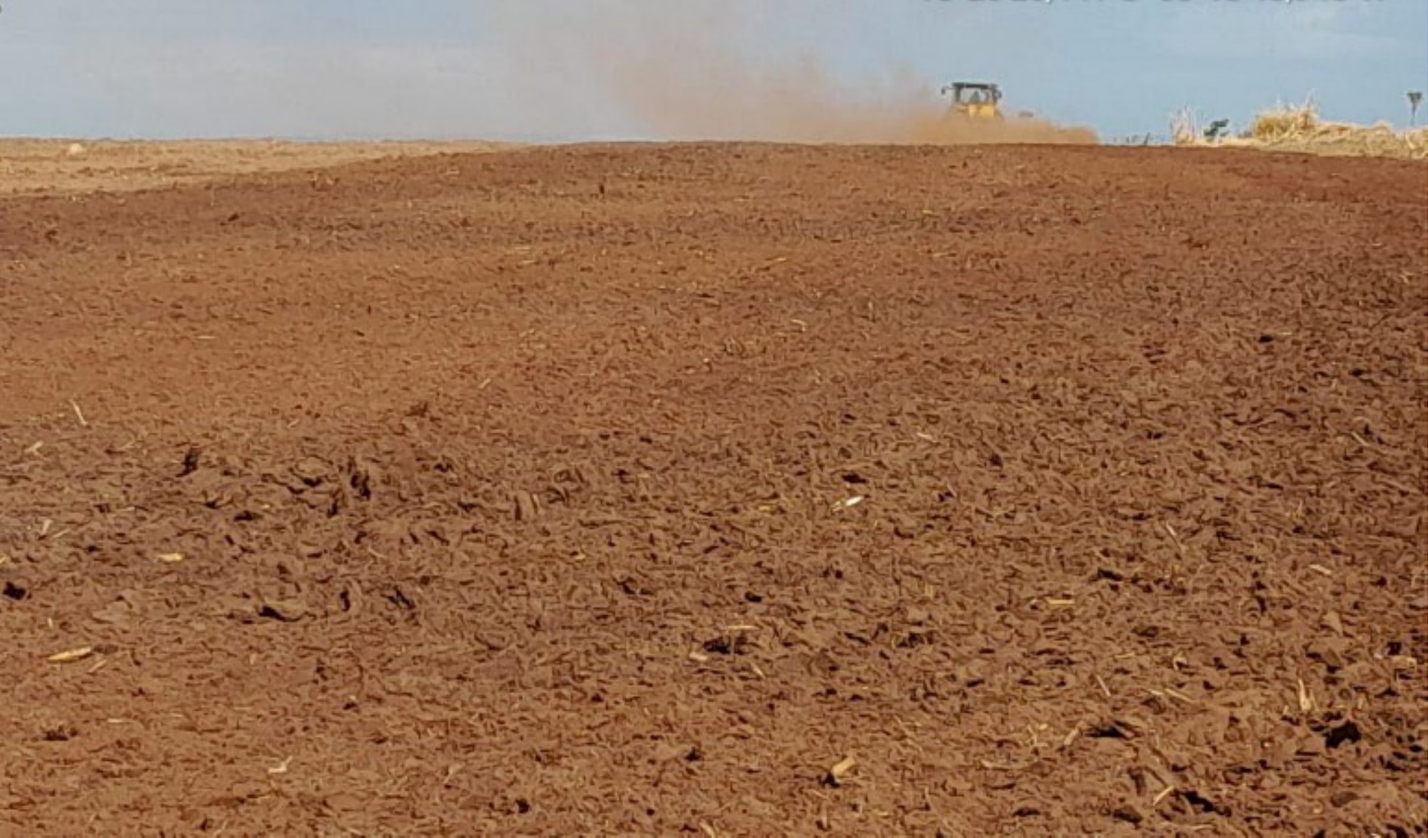
Soil with sludge after incorporation. *Source:* Author (2024).

Field preparation included subsoiling and intermediate harrowing to improve soil conditions and facilitate sludge incorporation. Crop residues present in the area were incorporated into the soil before sludge application. Subsequently, the sludge was uniformly distributed across the field and incorporated through harrowing and plowing operations, preventing surface accumulation and reducing the risk of nutrient losses through runoff. The incorporation process was repeated until the material was completely mixed with the soil profile, as illustrated in Figure 7.

Following application, the area was monitored by the technical team responsible for the operation to verify sludge incorporation efficiency and identify any signs of runoff or material accumulation. Additional harrowing operations were performed when necessary to ensure complete incorporation and minimize the potential transport of sediments and nutrients to nearby reservoirs, permanent preservation areas (PPAs), and watercourses. This management strategy was designed to promote the beneficial agricultural use of vinasse sludge while minimizing potential environmental impacts on soil and water resources.

### 2.4 Physicochemical Characterization of Vinasse Sludge

Prior to agricultural application, vinasse sludge samples were collected from the storage reservoir during cleaning operations and analyzed according to SEMADESC Resolution No. 001/2023. Representative samples were collected from the accumulated sludge, homogenized, properly packaged, and transported to an accredited laboratory for physicochemical characterization (Figure 5A,B).

The analyses included solids content, pH, electrical conductivity, nitrate, nitrite, ammoniacal nitrogen, total Kjeldahl nitrogen, total nitrogen, sodium, calcium, potassium, sulfate, and phosphate, aiming to assess both the agronomic potential and environmental implications of sludge application. The results, presented in Table 2, were used to support application rate calculations and evaluate compliance with environmental regulations.

**TABLE 2:** Analysis of sludge from reservoir number 02.

Analysis of sludge residue in accordance with SEMADESC 01/2023—RD2 no. 26518		
Parameters	Unit	RD2 no. 26518
Sampling date	dd/mm/yy	21/01/2021
Sampling time	time	16:30
Solids content	% w/w	48.2
pH (5% suspension)	—	4.06
Total solids	% w/w	48.2
Electrical conductivity (5% suspension)	mS/cm	< 1
Nitrate (as N)	mg/kg	< 10
Nitrite (as N)	mg/kg	5.83
Ammoniacal nitrogen	mg/kg	249
Total Kjeldahl nitrogen	mg/kg	28,400
Sodium	mg/kg	128
Calcium	mg/kg	649
Potassium	mg/kg	1950
Sulfate	mg/kg	783
Phosphate (as P)	mg/kg	468
Total nitrogen	mg/kg	28,406

*Source:* Prepared by the author (2023).

The analytical characterization provided the technical basis for the environmental assessment of vinasse sludge management and for the subsequent evaluation of potential effects on surface water quality within the monitored watershed.

### 2.5 Surface Water Sampling and Laboratory Analysis

Surface water monitoring was conducted from 2020 to 2023 at eight sampling points distributed throughout the watershed. Sampling campaigns were carried out twice a year, during the dry season (May) and the rainy season (November), resulting in a total of 64 samples. Water samples were collected at approximately 20 cm below the surface following the procedures described in the National Guide for Sample Collection and Preservation and the Standard Methods for the Examination of Water and Wastewater. Samples were preserved by refrigeration and, when required, by the addition of specific preservatives before transportation to the laboratory.

Field measurements of pH and dissolved oxygen were performed in situ using a calibrated multiparameter probe. Laboratory analyses followed standardized methods and included parameters related to nutrient enrichment, organic pollution, mineral composition, and general water quality, such as calcium, total coliforms, electrical conductivity, biochemical oxygen demand (BOD), chemical oxygen demand (COD), hardness, total phosphorus, manganese, nitrate, nitrite, total nitrogen, potassium, and turbidity. The analytical methods adopted for each parameter are presented in Table 3.

**TABLE 3:** Summary of the analytical method used for each parameter.

Parameters	Analytical method
Calcium	SM 3120 B
Total coliforms	SM 9223 B
Electrical conductivity	SM 2510 B
BOD (5 days)	SM 5210 B/SM 4500 OH
DQO	HACH 8000
Hardness	SM 2340 C
Total phosphorus (as P)	SM 4500 P D
Manganese	SM 3120 B
Nitrate (as N)	SM 4500 NO3 E
Nitrite (as N)	SM 4500 NO2 B
Total nitrogen	CÁLCULO
Dissolved oxygen	SM 4500 O H
pH	SM 4500 H + B
Potassium	SM 3120 B
Turbidity	SM 2130 B

*Source:* Author (2024).

The monitoring program generated a consistent dataset for the assessment of temporal and spatial variations in water quality and supported the evaluation of potential environmental impacts associated with the agricultural application of vinasse sludge.

### 2.6 Meteorological Conditions During the Monitoring Period

Meteorological conditions during the monitoring period were characterized using accumulated rainfall data for the months corresponding to the sampling campaigns. Precipitation data were used to support the interpretation of seasonal variations in surface water quality, particularly regarding the transport of sediments, nutrients, and dissolved constituents to watercourses.

The study region is characterized by marked seasonality, with rainfall concentrated during the wet season and reduced precipitation during the dry season. Variations in rainfall intensity and distribution may directly influence runoff generation, erosion processes, nutrient mobilization, and dilution effects in surface waters.

Table 4 presents the accumulated rainfall recorded during the sampling months (May and November) between 2020 and 2023. Considerable interannual variability was observed, with no precipitation recorded during some sampling campaigns, whereas November 2023 presented the highest accumulated rainfall (152.4 mm). Such variability provides important context for interpreting seasonal differences observed in the physicochemical parameters of the monitored watercourses.

**TABLE 4:** Accumulated rainfall during the sampling months from 2020 to 2023.

Year	May rainfall (mm)	November rainfall (mm)
2020	0.0	0.0
2021	90.4	0.0
2022	0.0	43.6
2023	57.0	152.4
Mean	36.9	49.0

*Source:* INMET https://portal.inmet.gov.br/dadoshistoricos.

The rainfall data indicate contrasting hydrological conditions among sampling periods, which may partially explain the seasonal variability observed for parameters such as turbidity, dissolved iron, and pH. Therefore, precipitation was considered an important environmental factor in the interpretation of water quality dynamics throughout the study period.

### 2.7 Statistical Analysis

Statistical analyses were performed using RStudio software to evaluate spatial and seasonal variations in surface water quality and the potential effects of vinasse sludge application. Data normality was assessed using the Shapiro–Wilk test.

For normally distributed variables, differences among monitoring points, sampling seasons, and application conditions were evaluated using analysis of variance (ANOVA) followed by Tukey's post hoc test. For non‐normally distributed variables, the Kruskal–Wallis test followed by Dunn's multiple comparison test with Bonferroni correction was applied.

To evaluate seasonal effects, all individual observations collected during the monitoring period (2020–2023) were grouped according to season (rainy or dry), and one‐way ANOVA was performed for each water‐quality parameter using season as the explanatory factor. The null hypothesis tested was that the mean value of each parameter did not differ between the rainy and dry seasons. The resulting *F* values and *p* values therefore represent the statistical significance of seasonal differences and do not correspond to comparisons among monitoring sites. Descriptive statistics (mean, standard deviation, minimum, and maximum) were calculated for all parameters, and a significance level of 5% (*p* < 0.05) was adopted.

The results were interpreted according to the environmental standards established by CONAMA Resolution No. 357/2005 and SEMADESC Resolution No. 001/2023.

## 3 Results and Discussion

This section presents the physicochemical characterization of vinasse sludge and the evaluation of surface water quality in the monitored watershed. The results were analyzed to assess spatial and seasonal variations in water quality parameters and to investigate potential effects associated with the agricultural application of vinasse sludge.

### 3.1 Characterization of Vinasse Sludge

The chemical characterization of vinasse sludge revealed the presence of substantial concentrations of nutrients, particularly total nitrogen (28,406 mg kg^−1^), ammoniacal nitrogen (249 mg kg^−1^), nitrite (583 mg kg^−1^), and phosphorus compounds (Table 2). These constituents indicate a considerable agronomic potential for soil amendment and nutrient recycling in sugarcane production systems (Christofoletti et al. 2013; Mahapatra et al. 2024).

Despite its potential agricultural benefits, some characteristics of the sludge require environmental attention. The elevated concentrations of nitrogen compounds may increase the risk of nutrient transport to adjacent water bodies through surface runoff or subsurface flow, potentially contributing to eutrophication processes and oxygen depletion in aquatic ecosystems. In addition, the acidic pH (4.06) may enhance nutrient mobility and influence the solubility of trace elements in soils and surface waters (Fuess 2017; Ferreira et al. 2021).

The sludge also presented high total solids content, indicating a significant load of suspended and settleable materials that could increase turbidity if transported to nearby streams. Conversely, the low electrical conductivity values suggest a low immediate risk of salinization under the application conditions evaluated in this study. Overall, the results indicate that vinasse sludge contains valuable nutrients for agricultural use; however, its elevated nutrient and solids contents also indicate a potential environmental risk if repeated applications are not properly managed. Appropriate management practices and continuous environmental monitoring are therefore essential to minimize the transport of nutrients and suspended materials to adjacent water bodies. Moreover, the potential accumulation of nutrients in soils and their delayed release to surface waters should be considered when assessing the long‐term environmental sustainability of vinasse sludge application.

To assess the potential influence of vinasse sludge application on adjacent surface waters, monitoring points were strategically established upstream and downstream of the application areas, allowing the evaluation of potential changes in water quality along the drainage network (Figure 2).

### 3.2 Spatial Variation of Water Quality Parameters

The descriptive statistics and analysis of variance (ANOVA) revealed significant spatial variability among the monitored points for several water quality parameters (Table 5). The results indicate that local environmental conditions, land use, and proximity to agricultural and urban activities influenced the distribution of physicochemical characteristics in the evaluated watercourses.

**TABLE 5:** Descriptive statistics (mean ± standard deviation) of selected water quality parameters at the monitoring points (2020–2023) and results of ANOVA.

Parameter	P1	P2	P3	P4	P5	P6	P7	P8	*F* value	*p*
Turbidity	8.21 ± 3.09	8.00 ± 3.19	7.65 ± 2.57	4.84 ± 6.57	6.45 ± 2.12	7.95 ± 4.37	23.64 ± 27.43	5.95 ± 4.38	0.95	0.478
Calcium	0.77 ± 0.42	0.73 ± 0.52	1.16 ± 0.67	0.41 ± 0.32	0.95 ± 0.72	2.56 ± 0.99	2.86 ± 0.56	0.46 ± 0.31	19.59	< 0.001
Electrical conductivity	8.28 ± 1.45	16.61 ± 16.88	11.66 ± 1.55	5.39 ± 0.76	11.21 ± 1.43	28.59 ± 8.84	32.04 ± 3.89	5.41 ± 2.21	17.17	< 0.001
Total chromium	0.0076 ± 0.0070	0.0076 ± 0.0070	0.0075 ± 0.0067	0.0081 ± 0.0070	0.0088 ± 0.0070	0.0075 ± 0.0071	0.0075 ± 0.0071	0.0076 ± 0.0070	0.03	1.000
Dissolved iron	0.122 ± 0.091	0.158 ± 0.076	0.144 ± 0.050	0.065 ± 0.048	0.214 ± 0.082	0.219 ± 0.092	0.266 ± 0.158	0.166 ± 0.041	4.16	< 0.001
Magnesium	0.294 ± 0.128	0.318 ± 0.201	0.438 ± 0.200	0.147 ± 0.098	0.417 ± 0.188	1.157 ± 0.367	1.392 ± 0.253	0.178 ± 0.146	38.58	< 0.001
Manganese	0.0081 ± 0.0072	0.0208 ± 0.0291	0.0122 ± 0.0088	0.0068 ± 0.0063	0.0168 ± 0.0099	0.0160 ± 0.0069	0.0400 ± 0.0231	0.0098 ± 0.0060	4.21	< 0.001
Ammoniacal nitrogen	0.100 ± 0.000	0.100 ± 0.000	0.100 ± 0.000	0.100 ± 0.000	0.100 ± 0.000	0.104 ± 0.011	0.174 ± 0.103	0.088 ± 0.035	3.87	0.002
pH	6.50 ± 0.30	6.45 ± 0.37	6.65 ± 0.52	6.29 ± 0.44	6.57 ± 0.44	7.09 ± 0.79	6.99 ± 0.69	6.33 ± 0.36	2.57	0.023

*Note:* Values are presented as mean ± standard deviation (SD). *F* values and *p* values correspond to ANOVA results. Significant differences were considered at *p* < 0.05.

Among the monitored sites, points P6 and P7 consistently presented higher values for several parameters, including calcium, electrical conductivity, dissolved iron, magnesium, ammoniacal nitrogen, and pH. These results suggest a greater influence of anthropogenic activities in these locations compared with most upstream sites and the control sites (Fuess 2017).

Point P6 is located adjacent to sugarcane cultivation areas where vinasse and vinasse sludge have historically been applied. The higher concentrations observed at this site may reflect the combined influence of agricultural management practices, local soil characteristics, and natural hydrogeochemical processes operating within the watershed. Nevertheless, all measured values remained within the limits established by Brazilian environmental regulations, indicating no evidence of significant water quality impairment.

Interestingly, the highest concentrations for several parameters were observed at P7, a site not directly influenced by vinasse sludge application. This finding suggests that factors other than sludge disposal, including watershed position, urban influences, natural soil conditions, and hydrological connectivity, may play a more important role in controlling water quality patterns than the agricultural application of vinasse sludge itself. Similar patterns have been reported in mixed land‐use watersheds, where agricultural and urban activities jointly influence water quality and may obscure the contribution of individual sources (Liang et al. 2013; Nobre et al. 2020).

Although the present results suggest that factors such as watershed characteristics, land use, and hydrological connectivity exerted a stronger influence on water quality than vinasse sludge application during the monitoring period, these findings should be interpreted with caution. The absence of detectable impacts in surface waters does not necessarily indicate the absence of environmental risk. Previous studies have demonstrated that repeated applications of nutrient‐rich organic residues may promote the accumulation of legacy nutrients in soils, which can be mobilized and transported to aquatic ecosystems years after their application under favorable hydrological conditions. Therefore, delayed impacts on surface water quality cannot be excluded based solely on the monitoring period evaluated in this study.

The influence of soil characteristics should also be considered when interpreting the spatial patterns observed. The predominance of highly weathered tropical soils in the study area favors nutrient retention through adsorption processes, reducing the mobility of some elements. However, sandy soils occurring in portions of the watershed may facilitate the transport of dissolved nutrients and fine sediments, especially under conditions of intense rainfall and inadequate soil cover.

Nevertheless, these mechanisms were not directly evaluated in the present study and therefore should be regarded as plausible explanations rather than definitive evidence. Future investigations integrating soil analyses, groundwater monitoring, and hydrological connectivity assessments are needed to verify the relative contribution of these processes.

Soil properties and land management practices are recognized as key controls on nutrient transport and water quality responses at the watershed scale, often mitigating the transfer of agricultural contaminants to surface waters (Teixeira et al. 2005; Peyton et al. 2016; Fu et al. 2020).

Despite the spatial variability observed among monitoring points, dissolved iron concentrations remained below the limit established by CONAMA Resolution No. 357/2005 for Class 2 freshwaters (0.3 mg L^−1^), while pH values remained within the acceptable range of 6.0–9.0. These results indicate that the monitored watercourses‐maintained water quality conditions compatible with the applicable environmental standards throughout the study period. Nevertheless, compliance with current environmental standards during the monitoring period should not be interpreted as evidence that long‐term impacts cannot occur, particularly considering the potential accumulation and delayed mobilization of nutrients reported in previous studies.

Despite the higher concentrations observed at P6 and P7, most parameters remained below the thresholds established by CONAMA Resolution No. 357/2005 and the guidelines of SEMADESC (BRASIL 2005; MATO GROSSO DO SUL 2023). These findings indicate that no statistically detectable deterioration in surface water quality attributable to vinasse sludge application was observed during the monitoring period. However, these results should not be interpreted as evidence that long‐term environmental impacts are absent. The accumulation of legacy nutrients in soils, their delayed mobilization under changing hydrological conditions, and potential transport to downstream aquatic ecosystems remain plausible processes that require long‐term investigation. Consequently, continued environmental monitoring and future studies integrating soil, groundwater, and surface‐water assessments are necessary to evaluate the long‐term sustainability of repeated vinasse sludge application.

### 3.3 Seasonal Variation of Water Quality Parameters

**FIGURE 8:**
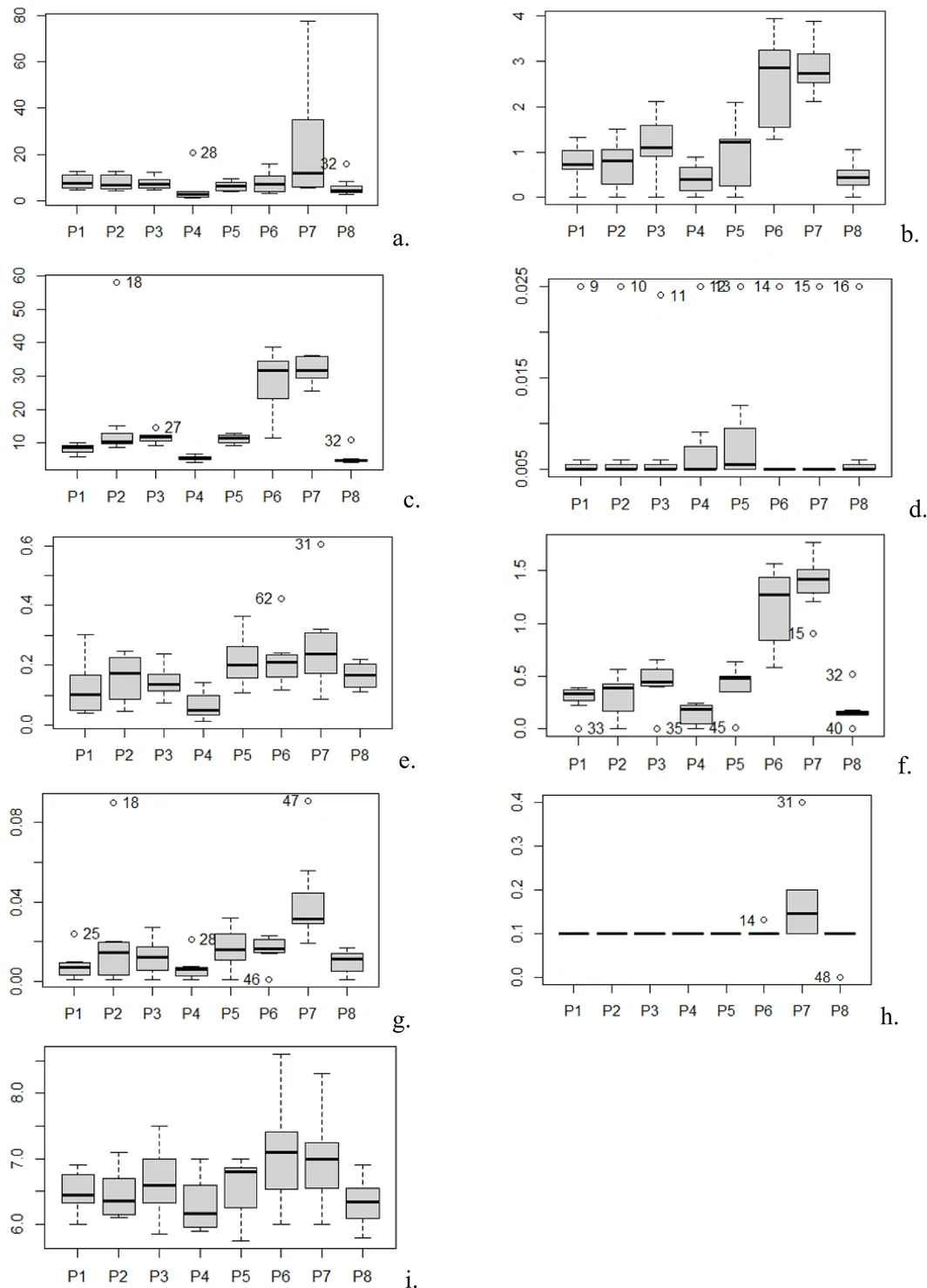
Boxplots showing the seasonal variation of selected water quality parameters between the rainy and dry seasons (2020–2023). Legends: (a) turbidity, (b) calcium, (c) electrical conductivity, (d) total chromium, (e) dissolved iron, (f) magnesium, (g) manganese, (h) ammoniacal nitrogen, and (i) pH. Significant seasonal differences were observed for turbidity, total chromium, dissolved iron, and pH (*p* < 0.05).

Seasonal variation in water quality was evaluated by comparing the rainy and dry periods using descriptive statistics, boxplots (Figure 8), and analysis of variance (Table 6). The results demonstrated that most parameters exhibited relatively stable behavior throughout the monitoring period, although some variables showed statistically significant seasonal differences.

**TABLE 6:** Seasonal comparison of water quality parameters between the rainy and dry seasons considering all individual observations collected during the monitoring period (2020–2023).

Parameter	Rainy season (mean ± SD)	Dry season (mean ± SD)	*F* value	*p*
Turbidity (NTU)	6.02 ± 2.79	12.15 ± 15.27	4.99	0.029*
Calcium (mg L^−1^)	1.24 ± 1.11	1.23 ± 1.01	0.00	0.971
Electrical conductivity (mS cm^−1^)	16.16 ± 13.68	13.64 ± 9.31	0.74	0.393
Total chromium (mg L^−1^)	0.0055 ± 0.0013	0.0101 ± 0.0087	8.85	0.004*
Dissolved iron (mg L^−1^)	0.141 ± 0.069	0.120 ± 0.120	5.28	0.025*
Magnesium (mg L^−1^)	0.565 ± 0.532	0.520 ± 0.438	0.13	0.716
Manganese (mg L^−1^)	0.0152 ± 0.0164	0.0174 ± 0.0180	0.27	0.606
Ammoniacal nitrogen (mg L^−1^)	0.100 ± 0.000	0.116 ± 0.062	2.17	0.146
pH	6.79 ± 0.61	6.43 ± 0.43	7.46	0.008*

*Note:* Values are presented as mean ± standard deviation (SD). Seasonal comparisons were performed using one‐way analysis of variance (ANOVA), considering season (rainy or dry) as the explanatory factor and all individual observations collected during the monitoring period. The reported *F* and *p* values test the null hypothesis that the mean value of each parameter is equal between seasons. Significant differences (*p* < 0.05) were observed for turbidity, total chromium, dissolved iron, and pH.

* Significant differences at *p* < 0.05.

Seasonal differences were evaluated using one‐way ANOVA considering season (rainy versus dry) as the explanatory factor and all individual observations collected during the monitoring period. The results are summarized in Table 7.

**TABLE 7:** Descriptive statistics of selected water quality parameters in areas with and without vinasse sludge application (2020–2023).

Parameter	Without application (mean ± SD)	With application (mean ± SD)	*F* value	*p*
Turbidity (NTU)	17.68 ± 23.82	7.66 ± 4.13	6.07	0.018*
Calcium (mg L^−1^)	1.59 ± 1.23	0.95 ± 0.87	3.95	0.053
Electrical conductivity (μS cm^−1^)	19.18 ± 14.52	14.35 ± 11.39	1.41	0.242
Total chromium (mg L^−1^)	0.0084 ± 0.0078	0.0087 ± 0.0074	0.01	0.912
Dissolved iron (mg L^−1^)	0.228 ± 0.132	0.157 ± 0.082	4.90	0.032*
Magnesium (mg L^−1^)	0.771 ± 0.652	0.424 ± 0.378	5.15	0.028*
Manganese (mg L^−1^)	0.0257 ± 0.0258	0.0124 ± 0.0156	4.67	0.036*
Ammoniacal nitrogen (mg L^−1^)	0.141 ± 0.072	0.101 ± 0.015	7.45	0.009*
pH	6.62 ± 0.63	6.66 ± 0.41	0.04	0.840

*Note:* Values are presented as mean ± standard deviation (SD). Monitoring points P1–P6 were classified as areas with vinasse sludge application, whereas P7 and P8 were considered reference areas without application. Comparisons between groups were performed using one‐way ANOVA. *F* values and *p* values refer to the comparison between application conditions. Significant differences are indicated by * (*p* < 0.05).

* Significant differences at *p* < 0.05.

Turbidity presented significantly higher values during the dry season (12.15 NTU) compared with the rainy season (6.02 NTU) (*p* = 0.029). The boxplots also revealed greater variability at P7, indicating that local factors may contribute to episodic increases in suspended solids independently of seasonal effects. Although increased precipitation is often associated with sediment transport, the lower turbidity observed during the rainy period may be related to dilution effects and greater streamflow volumes. Conversely, during the dry season, reduced water availability may concentrate suspended particles within the water column (Fu et al. 2020). Nevertheless, turbidity values remained below the limits established by CONAMA Resolution No. 357/2005 throughout the study period.

Total chromium also showed significant seasonal variation (*p* = 0.004), with slightly higher concentrations during the dry season. Although statistically significant, chromium concentrations remained very low throughout the study period and showed limited variability among monitoring points; all measured values remained substantially below the regulatory threshold of 0.05 mg L^−1^, indicating low environmental risk associated with chromium contamination (Ibrahim et al. 2021).

Dissolved iron concentrations differed significantly between seasons (*p* = 0.025), with higher mean values observed during the rainy period. Higher dissolved iron concentrations were particularly evident at P6 and P7, suggesting the influence of local watershed characteristics in addition to seasonal rainfall effects. This pattern may be associated with increased soil erosion, transport of fine particles, and mobilization of iron‐rich materials during rainfall events. Similar seasonal responses have been reported in tropical watersheds where precipitation enhances sediment and nutrient transport from surrounding soils into surface waters (Rodrigues et al. 2018).

The pH values also varied significantly between seasons (*p* = 0.008), with slightly higher values during the rainy period. The highest pH values were observed at P6 and P7, although all measurements remained within the regulatory limits established for Class 2 freshwaters. Although statistically significant, the observed variation was relatively small and remained within the acceptable range established for freshwater bodies under Brazilian environmental legislation.

In contrast, calcium, electrical conductivity, magnesium, manganese, and ammoniacal nitrogen did not show significant seasonal differences (*p* > 0.05). For these parameters, spatial differences among monitoring sites were more pronounced than seasonal variations, suggesting stronger control by local environmental and land‐use conditions.

Overall, the seasonal analysis indicates that rainfall influenced the temporal dynamics of selected water quality parameters, particularly turbidity, dissolved iron, total chromium, and pH. However, the stronger differences observed among monitoring sites suggest that watershed characteristics, land use, and local hydrogeochemical conditions exerted greater control over water quality than seasonal climatic variability. Similar patterns have been reported in agricultural and mixed‐use tropical watersheds where spatial heterogeneity frequently overrides seasonal effects on surface‐water chemistry (Mello et al. 2018).

### 3.4 Effect of Vinasse Sludge Application on Surface Water Quality

To evaluate the potential influence of vinasse sludge application on adjacent water resources, water quality parameters were compared between monitoring sites located in areas without sludge application and those subjected to agricultural sludge disposal (Table 7).

Overall, the results did not indicate substantial deterioration of surface water quality associated with sludge application. Most evaluated parameters showed similar values between the two groups, while some variables exhibited lower concentrations in areas receiving sludge. These findings indicate that water quality conditions were generally similar between areas with and without sludge application, suggesting no clear evidence of adverse effects associated with the management practices adopted during the study period.

Turbidity showed lower mean values in areas with sludge application (7.66 NTU) compared with areas without application (17.68 NTU). Likewise, dissolved iron concentrations decreased from 0.228 mg L^−1^ in nonapplication areas to 0.157 mg L^−1^ in application areas. Similar reductions were observed for calcium, magnesium, manganese, ammoniacal nitrogen, and electrical conductivity. These patterns do not provide evidence that sludge application was associated with increased sediment transport or elevated concentrations of dissolved constituents in the monitored streams (Liang et al. 2013; Stephen et al. 2024; Mahapatra et al. 2024).

An important observation is that some of the highest mean values for turbidity, electrical conductivity, dissolved iron, magnesium, manganese, and ammoniacal nitrogen were recorded at P7, a reference site without direct sludge application. Therefore, P7 should not be interpreted as a true control site, since it may still be influenced by diffuse upstream land uses, surface runoff, and other watershed processes. This finding suggests that spatial variability in water quality was influenced by factors beyond sludge disposal, including watershed characteristics, hydrological conditions, and surrounding land‐use patterns. Consequently, the observed differences among monitoring points cannot be attributed exclusively to the agricultural use of vinasse sludge.

Likewise, the absence of pronounced differences between monitoring sites P5 and P8 suggests that local environmental conditions and watershed characteristics may exert a stronger influence on water quality than the presence or absence of direct sludge application. Although P5 is located within the sludge application area and P8 represents a reference site, the similarity observed between these monitoring points reinforces the importance of considering multiple environmental drivers, including soil properties, hydrological connectivity, and surrounding land‐use patterns, when interpreting spatial variations in monitored parameters.

Total chromium concentrations remained virtually unchanged between the two groups, with mean values of 0.0087 and 0.0084 mg L^−1^ for areas without and with sludge application, respectively. Furthermore, all chromium concentrations remained well below the maximum limit established by CONAMA Resolution No. 357/2005, indicating no evidence of contamination associated with sludge disposal practices.

The pH values also showed only minor variation between the evaluated conditions, with mean values of 6.66 and 6.62 in areas without and with sludge application, respectively. These values remained within the acceptable range for freshwater ecosystems and suggest that sludge application did not significantly alter the acid–base balance of the monitored streams.

The observed results may also be related to the predominance of highly weathered Latosols and other tropical soils in the study area, which have a considerable capacity to retain nutrients and adsorb dissolved compounds. These characteristics reduce nutrient mobility and limit their transport to adjacent water bodies. In addition, the implementation of sludge application practices in accordance with state environmental regulations likely contributed to minimizing potential off‐site impacts.

Although the chemical characterization of the sludge revealed the presence of nutrients and compounds that could potentially affect water quality, the monitoring data did not indicate significant adverse effects on the evaluated streams during the study period. Nevertheless, continued monitoring remains important, particularly at points P6 and P7, where higher concentrations of some parameters were observed and where cumulative effects may become more evident over longer time scales (Carrilho et al. 2016; Chukalla et al. 2024).

Monitoring site P6 consistently exhibited higher values for several water‐quality parameters, suggesting greater environmental sensitivity within the watershed. This behavior may reflect the combined influence of local topography, drainage characteristics, seasonal runoff, and surrounding land‐use practices rather than the direct effect of vinasse sludge application alone. These findings indicate that hydrological connectivity and local watershed processes may play an important role in controlling water‐quality variability at this site.

Overall, the comparison between areas with and without sludge application did not reveal consistent patterns of water quality degradation associated with vinasse sludge disposal. The similarity of most monitored parameters between the two groups, together with the occurrence of elevated values at reference sites, suggests that local watershed characteristics exerted a stronger influence on water quality than sludge application during the study period. Nevertheless, continued monitoring remains essential to identify potential cumulative effects and to support adaptive environmental management strategies.

### 3.5 Environmental Implications

The results obtained in this study provide important insights into the environmental implications of vinasse sludge application in agricultural areas. Although the physicochemical characterization of the sludge revealed the presence of nutrients and compounds with the potential to affect water quality, the monitoring of adjacent surface waters did not indicate substantial environmental impacts during the evaluated period.

Although all monitored parameters remained within the limits established by CONAMA Resolution No. 357/2005 and the environmental guidelines adopted by SEMADESC, compliance with regulatory thresholds alone should not be interpreted as evidence of the absence of environmental impacts. Water‐quality responses are influenced by multiple watershed processes, including soil characteristics, hydrological connectivity, land‐use patterns, and seasonal climatic variability. Therefore, the observed spatial and temporal patterns should be interpreted within this broader environmental context (BRASIL 2005, 2020).

The spatial and seasonal analyses, together with the comparison between areas with and without sludge application, did not reveal consistent patterns of surface water degradation associated with the agricultural use of vinasse sludge. Overall, water quality remained stable throughout the monitoring period, suggesting that the adopted management practices were compatible with the maintenance of environmental quality in the monitored streams.

Particular attention should be given to points P6 and P7, which consistently exhibited higher values for several parameters, including turbidity, electrical conductivity, dissolved iron, magnesium, ammoniacal nitrogen, and pH. These results indicate greater environmental sensitivity at these locations and highlight the influence of local watershed characteristics and land‐use conditions on water quality dynamics. Although concentrations remained within regulatory limits, these sites should be prioritized in future monitoring programs (Rolim et al. 2013; Mello et al. 2018).

The absence of detectable impacts during the monitoring period should not be interpreted as the absence of environmental risk. Nutrient accumulation in soils, delayed nutrient mobilization, changes in land use, and long‐term watershed processes may produce delayed responses that are not immediately reflected in surface water quality (Ferreira et al. 2021; Frota 2023). Furthermore, the mechanisms proposed in the present study, including nutrient retention by highly weathered tropical soils and attenuation within the watershed, should be regarded as hypotheses that require confirmation through long‐term monitoring, soil analyses, and hydrological investigations. Future studies should therefore evaluate legacy nutrient accumulation, delayed transport processes, and the hydrological connectivity between treated fields and receiving water bodies to verify the long‐term environmental implications of vinasse sludge application.

Overall, the findings indicate that vinasse sludge can be reused as an agricultural amendment without measurable deterioration of surface water quality when application practices comply with environmental regulations and are compatible with local soil and watershed conditions. This reinforces the potential of vinasse sludge valorization within a circular economy framework, while highlighting the importance of long‐term environmental monitoring to ensure the sustainable management of water resources (Fuess 2017; German et al. 2020).

## 4 Conclusion

The physicochemical characterization of vinasse sludge revealed the presence of nutrients with agronomic value, particularly nitrogen, potassium, calcium, and phosphorus, highlighting its potential for agricultural reuse. At the same time, the occurrence of compounds that may affect water quality reinforces the need for appropriate management practices and regulatory compliance.

Surface water monitoring conducted between 2020 and 2023 showed that all evaluated physicochemical parameters remained within the limits established by CONAMA Resolution No. 357/2005. Spatial and seasonal analyses, as well as comparisons between areas with and without sludge application, did not reveal consistent evidence of surface water quality degradation associated with the agricultural use of vinasse sludge during the monitoring period.

Although some variability was observed, particularly at monitoring points P6 and P7, the recorded concentrations remained within environmental standards. These findings suggest that local watershed characteristics, land‐use conditions, and hydrogeochemical processes may have influenced the observed spatial patterns more strongly than vinasse sludge application under the conditions evaluated. However, these mechanisms were not directly investigated in the present study and therefore should be regarded as plausible explanations rather than definitive evidence (Benke et al. 1999; Mello et al. 2018).

Overall, the results indicate that no measurable impacts of vinasse sludge application on adjacent surface waters were detected under the environmental conditions and monitoring period evaluated in this study. However, these findings should not be interpreted as evidence that long‐term environmental impacts cannot occur. Previous studies have demonstrated that repeated applications of nutrient‐rich residues may promote the accumulation of legacy nutrients in soils, which can be mobilized under changing hydrological conditions and affect surface waters over longer time scales.

Therefore, continued environmental monitoring remains essential to evaluate potential cumulative effects and to support the sustainable management of water resources and agro‐industrial residues. Future studies integrating soil, groundwater, and hydrological connectivity assessments are recommended to better understand the long‐term environmental behavior of vinasse sludge application.

## Author Contributions

**Carlos Frederico de Souza Castro:** supervision, resources, data curation, validation, methodology, conceptualization, project administration, formal analysis, writing – review and editing. **Wagner Tavares Dos Santos:** conceptualization, investigation, methodology, writing – review and editing, formal analysis, validation. **Célsio Assane:** writing – original draft, writing – review and editing, data curation. **Bruno de Oliveira Costa Couto:** writing – review and editing, visualization, supervision, methodology, validation.

## Funding

This research received no specific funding from public, commercial, or not‐for‐profit funding agencies. The authors acknowledge the Coordenação de Aperfeiçoamento de Pessoal de Nível Superior (CAPES), Brazil, for providing support toward the article processing charge (APC) associated with the publication of this article.

## Conflicts of Interest

The authors declare no conflicts of interest.

## Data Availability

The data that support the findings of this study are available from the corresponding author upon reasonable request.
